# Tissue factor as a new target for CAR-NK cell immunotherapy of triple-negative breast cancer

**DOI:** 10.1038/s41598-020-59736-3

**Published:** 2020-02-18

**Authors:** Zhiwei Hu

**Affiliations:** Department of Surgery, Division of Surgical Oncology, The Ohio State University Wexner Medical Center and The OSU James Comprehensive Cancer Center, Columbus, OH 43210 USA

**Keywords:** Breast cancer, Immunotherapy, Tumour immunology

## Abstract

Triple-negative breast cancer (TNBC), representing ~15% of globally diagnosed breast cancer, is typically an incurable malignancy due to the lack of targetable surface targets for development of effective therapy. To address the unmet need for TNBC treatment, we recently determined that tissue factor (TF) is a useful surface target in 50–85% of patients with TNBC and developed a second-generation TF-targeting antibody-like immunoconjugate (called L-ICON) for preclinical treatment of TNBC. Using the chimeric antigen receptor (CAR) approach, here we develop and test TF-targeting CAR-engineered natural killer (TF-CAR-NK) cells that co-express CD16, the Fc receptor (FcγIII) to mediate antibody-dependent cellular toxicity (ADCC), for a preclinical assessment of immunotherapy of TNBC using TF-CAR-NK cell as single agent therapy and in combination with L-ICON. Our preclinical results demonstrate that TF-CAR-NK cells alone could kill TNBC cells and its efficacy was enhanced with L-ICON ADCC *in vitro*. Moreover, TF-CAR-NK cells were effective *in vivo* for the treatment of TNBC in cell line- and patient’s tumor-derived xenograft mouse models. Thus, this study established the proof of concept of targeting TF as a new target in CAR-NK immunotherapy for effective treatment of TNBC and may warrant further preclinical study and potentially future investigation in TNBC patients.

## Introduction

The adoptive transfer of chimeric antigen receptor (CAR)-expressing T and natural killer (NK) cells represents a novel cancer immunotherapy approach. The concept of the CAR is based upon the idea of expressing novel receptors on the T or NK cell surface that would enable the T and NK cell to identify corresponding antigens on the surface of a target cell. The basic CAR construct consists of an extracellular antigen-recognition domain, usually single-chain antibody variable fragments (scFv), attached to an extracellular spacer domain, a transmembrane domain of CD28 and a signaling cytoplasmic domain such as 4-1BB (CD137), OX40 (CD134), DAP10, ICOS and CD3zeta chain (CD3ζ). The most advanced application is the use of CAR-T cells targeting CD19, a surface antigen on B cell malignancies, which has demonstrated antitumor efficacy in patients with these cancers^1^. However, early-phase clinical trials of CAR T therapy also showed that this treatment is frequently associated with side effects^1^, some even causing life-threatening toxicity^2^, partly due to the fact that current CAR targets are expressed by both the malignant cells and normal cells, such as CD19, which is also expressed by normal B cells throughout the B cell lineage^1^. Also, one of the major challenges in CAR therapy is the heterogeneity of tumor antigens, for instance, antigen loss or low antigen density on the cancer cells^3^. To overcome these challenges, it would be ideal to develop CAR T or CAR NK cells that could target a surface molecule that is commonly yet selectively expressed by several major tumor compartments, including but not limited to, the cancer cell, cancer stem cell (CSC) and tumor vascular endothelial cell (VEC) (to achieve stronger efficacy, eliminate metastasis and recurrence), whereas it is not expressed on normal peripheral blood mononuclear cells (such as B, T, NK cells, monocytes, etc.) and resting vascular endothelial cells (to avoid or reduce side effects).

Triple-negative breast cancer (TNBC) represents ~15% of globally diagnosed breast cancer^4–6^. TNBC is so-called because in this type of cancer, the tumor cells lack expression of estrogen receptor, progesterone receptor and the cell surface protein epidermal growth factor receptor (HER2)^5,7–12^, all of which are therapeutic targets on non-TNBC cells. Despite current preclinical and early clinical investigation of combination chemotherapy^13^, there are currently no molecularly targeted therapies approved for TNBC and therefore, TNBC is one of the most difficult-to-treat malignancies, making it, in most cases, an incurable disease.

To identify a targetable surface molecule for TNBC therapy, we demonstrated that tissue factor (TF) is a novel, common yet selective oncotarget on the TNBC cancer cells (50% to 85% of 161 patients with TNBC) and tumor VECs (64% of 14 patients with TNBC)^14^. In addition, our laboratory reported that TF is a novel oncotarget for CSCs, which were isolated from human breast, lung and ovarian cancer cell lines including TNBC, tumor xenografts and patients with breast cancer^15^. We also found that CSCs, including those isolated from human TNBC lines and breast cancer patients, did not develop resistance to TF-targeting therapy^15^. Moreover, a previous study reported that TF was also highly expressed on the breast cancer cells and selectively on tumor vascular endothelial cells in patients with non-triple negative breast cancer (non-TNBC)^16^. Thus, we believe that TF is a common yet specific oncotarget for three important tumor compartments, i.e., cancer cells, tumor VECs and CSCs, in both TNBC and non-TNBC. So far, there is no published study of TF-targeting human CAR-NK cells.

TF, also known as coagulation factor III and CD142, is a 47-kDa membrane-bound cell surface receptor^17–19^. Its best known function involves the initiation of blood coagulation upon disruption of vessel wall integrity^18,19^. Under physiological conditions, TF is not expressed on peripheral blood lymphocytes and quiescent vascular endothelial cells (the inner layer) of normal blood vessels in normal tissues and organs^20–23^. Besides its role as the primary initiator of coagulation, TF is also a modulator of pathological angiogenesis^24–26^. We and our collaborators along with other groups have identified TF as an angiogenesis-specific receptor on VEGF-stimulated angiogenic microvascular endothelial models *in vitro* as well as *in vivo* in angiogenic VECs (the inner layer) of the pathological neovasculature of endometriosis, age-related macular degeneration (AMD) and solid cancers, including melanoma^27,28^, lung cancer^29^ and breast cancer^29^, and from tumor xenografts in mice and breast cancer tissues from patients^14,16^. In cancer, TF is highly expressed on the cancer cells in many types of solid cancers^14,23,30–32^, acute myeloid and lymphoblastic leukemia (AML and ALL) and sarcoma^23,32^ as well as in Hodgkin’s lymphoma^33^ and multiple myeloma (MM, TF detected in 10 out of 18 patients with MM and 3 MM lines)^34^.

To target TF for antibody immunotherapy, Hu and Garen developed the first TF-targeting immunotherapy agent (called ICON)^27,28,35^. ICON is a chimeric antibody-like homodimer immunoconjugate (210 kDa)^36^ that consists of murine or human fVII full-length peptide (406 amino acid residues, aa) fused to the Fc region of IgG1^27,28,35,37^. The procoagulant effects of ICON-encoded zymogen fVII have been significantly eliminated via targeted mutation of the lysine reside at position 341 (K341A)^35^, but was not completely depleted (5% of activated fVII, fVIIa)^14^. Our lab recently improved it to a second-generation ICON (called L-ICON or L-ICON1) by removing the procoagulant heavy chain of fVII from ICON, resulting in a >50% reduction in molecular mass (92.5 kDa), complete depletion of procoagulant activity^14^ and higher binding activity and antibody-dependent cell-mediated cytotoxicity (ADCC) to TNBC cells than the original ICON. It is well documented that NK cells are crucial as CD16+ ADCC effector cells for the efficacy of antibody immunotherapy using ICON^36^, L-ICON^14^ or other therapeutic antibody^38^. However, NK cells are often impaired in cancer patients^38^, including patients with breast cancer^39,40^. NK impairment in cancer patients could reduce the therapeutic efficacy of ICON, L-ICON and antibody immunotherapy.

To address the unmet need for TNBC treatment and to overcome NK impairment and enhance L-ICON efficacy via ADCC killing mechanism, I constructed TF-targeting CAR NK cells using NK92MI (ATCC), an interleukin-2 (IL-2) independent human NK cell line^41^ as a NK cell model. The NK92MI line has been stably transfected for co-expression of full length CD16 (fCD16) in my laboratory prior to lentiviral transduction. After verifying their expression of CAR and CD16, I evaluated their direct cytotoxicity and their ability to mediate L-ICON ADCC against TNBC cells *in vitro* and therapeutic efficacy and safety *in vivo* in orthotopic mouse models of TNBC cell line-derived and patient’s tumor-derived xenografts (CDX and PDX).

## Results

### Design and expression of TF-targeting CAR monomer and dimer on NK cell line

**Figure 1:**
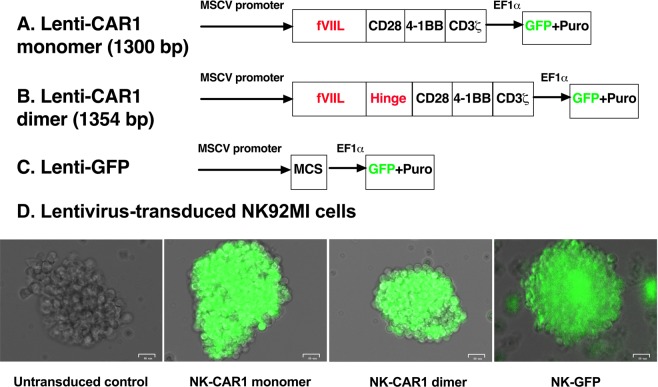
Constructs of TF-targeting CAR1 monomer and dimer and generation of stable CAR1-NK cell lines. (**A**,**B**) Lentivirus encoding CAR1 monomer and dimer (GenBank accession no. MF806378 and MF806379). fVIIL: Factor VII light chain. Hinge: Hinge region of human IgG1 (GenBank accession no. KX760097). GFP: Green fluorescence protein. Puro: Puromycin resistant gene. The only difference between CAR1 monomer and dimer is that the dimer construct contains an IgG1 hinge region, in which cysteine residues can form disulfide bonds to form a homologous dimer. (**C**) Control lentivirus does not encode CAR but only GFP (Lenti-GFP). MCS: Multi-cloning sites. (**D**) Generation of stable TF-targeting CAR1-NK cell lines. NK-CAR1 monomer and dimer: Lentivirus encoding CAR1 monomer and dimer-transduced NK92MI/fCD16 stable cell lines under selection of 2.5 μg/ml puromycin. NK-GFP: Lentivirus encoding GFP (Lenti-GFP)-transduced NK92MI/fCD16 cells were generated and used as a lentivirus-transduced control. Untransduced parental NK92MI cells did not express GFP and were used as a GFP-negative untransduced control. Photos were taken using bright field and green channels and were merged under Zeo Cell Imager (Bio-Rad). Scale bars: 25 μm.

The TF-targeting CARs for this study consist of Kozak sequence and human fVII light chain (including signal peptide sequence and 152 mature fVII light chain amino acid residues, aa) as the TF-targeting domain, without or with a hinge region of human IgG1 (16 amino acid residues containing three cysteines, AEPKSCDKTHTCPPCP), followed by human CD28 transmembrane and cytoplasmic domains and then by human cytoplasmic domains of 4-1BB and CD3ζ (Fig. 1A,B and Supplementary Fig. S1), named TF-targeting CAR1 monomer and dimer (TF-CAR1). The cDNA sequences of CAR1 monomer and dimer were confirmed by Sanger DNA sequencing (Supplementary Fig. S1) and have been deposited at GenBank (accession no. MF806378 and MF806379). The TF-CAR1 cDNAs are encoded in lentiviral vectors under MSCV promoter (Lenti-CAR1 monomer and dimer). To visualize and monitor Lentivirus-transduced cells *in vitro* and potentially *in vivo*, these lentiviral vectors also encode green fluorescence protein (GFP) under a separate promoter (EF1α), whereas a control lentiviral vector only encodes GFP without encoding a CAR construct (Lenti-GFP) (Fig. 1C).

To ensure that the pCDH-based lentivirus (in the supernatant or concentrated by PEG-it reagent) contains infectious particles, 293AD cells, a different virus packaging cell line from 293TN cells used for making lentivirus in this study, were infected by the pCDH-based lentivirus encoding both GFP and CAR1 construct (dimer or monomer) (Fig. 1A–C) in supernatant or concentrated by PEG-it reagent (SBI). Supplementary Fig. S2 showed that after transduction with lentivirus either in SNT or concentrated vector, 293AD cells expressed GFP, whereas uninfected 293AD cells were negative for GFP expression. The GFP expression results suggested that both the supernatants and PEG-it concentrated samples contained infectious virus.

After verifying that the pCDH-based lentivirus productions contained infectious virus, I attempted to transduce NK and T cells to generate TF-targeting CAR-NK cells for this study and potentially CAR-T cells for a separate study in the future. NK and T cells are, in general, difficult to transduce by virus. To ensure transduction of CAR constructs into NK and T cells, we subcloned the CAR1 dimer and monomer cDNAs into the multicloning site (MCS) of lentiviral plasmid pCDH (Fig. 1A–C and Supplementary Fig. S1), which has successfully transduced human NK line NK92^42^.

In this study, I chose NK92MI (ATCC CRL-2408) as a NK cell model to generate CAR1-NK cells. Unlike its parental line NK92, NK92MI is IL-2 independent^41^. Both NK92 and NK92MI lines express granzyme B and perforin^43^ (necessary for their cytotoxicity) but have lost CD16^44^, the Fc receptor (FcγIII) for NK cells to mediate IgG1 and IgG3 antibody-dependent cellular toxicity (ADCC). In order for NK92MI cells to regain the ability to mediate ADCC, prior to lentivirus transduction I transfected NK92MI with plasmid pcDNA3.1(+) encoding full length human CD16 (fCD16) to generate a CD16+ NK cell line as effector cells to potentially mediate ICON, L-ICON and antibody ADCC. The resulting cells, designated as NK92MI/fCD16 (with G418 resistance from pcDNA3.1 vector), were then transduced by Lenti-CAR1 dimer and monomer viruses, which also encode GFP (acquiring additional resistance to puromycin from lentiviral vector. Fig. 1). To test the transduction efficiency into T cells, a pilot study was performed using a Jurkat-based T cell line (ADCC effector cells, Promega) as a T cell model.

Supplementary Fig. S3 showed that both the NK92MI/fCD16 and Jurkat T cell line could be transduced by lenti-CAR1 dimer, -CAR1 monomer and a control lenti-GFP virus. Based on the GFP positive cell percent, however, the lentivirus transduction efficiency was estimated at about 10% in both NK92MI and Jurkat-based T cell lines on day 2 after lentivirus transduction. Another factor that could contribute to relatively low transduction efficiency was that the lentivirus titers in the supernatants and in the concentrated samples were not determined so that the MOI (multiplicity of infection) might not be ideal. To get a pure population of selection marker (puromycin) resistant NK cells, puromycin was added on day 3 after lentivirus transduction. Untransduced NK92MI/fCD16 cells were killed about one week after selection with 5 μg/ml puromycin, whereas lentivirus-CAR1 dimer, -monomer and -GFP transduced NK92MI/fCD16 cells survived and were all positive for GFP expression on day 13 (Supplementary Figs. S4 and 1D). These results indicated that lentivirus has successfully transduced NK92MI cells. These lentivirus-stably transduced NK92MI/fCD16 cells were abbreviated hereafter as NK-CAR1 dimer and monomer, and NK-GFP, respectively.

**Figure 2:**
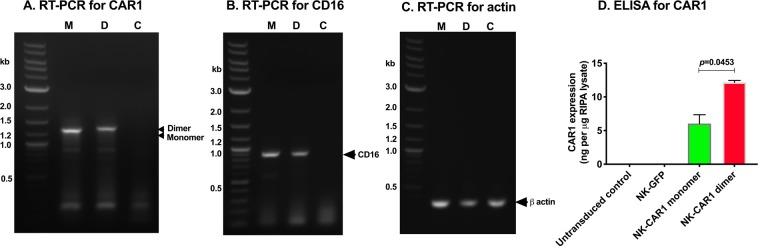
Expression of CAR1 and CD16 in NK92MI cells by RT-PCR and ELISA. (**A**–**C**) Total RNA was extracted by Trizol reagents from NK92MI/fCD16 cells stably transfected with CAR1 monomer (labeled as M in panels (A–C) and NK-CAR1 monomer in panel (D) or dimer (labeled as D in panels (A–C) and NK-CAR1 dimer in panel (D). Untransduced parental NK92MI cells were used as a negative control (**C**) for CAR1 and CD16. (**A**) CAR1. (**B**) CD16. (**C**) Beta actin was assayed as a loading control for total RNAs. Representative photos from two independent experiments. Quick-Load 2-Log DNA ladder (New England Biolabs) was used as DNA markers (0.1–10 Kilobases, Kb). (**D**) Sandwich ELISA assay using paired anti-human fVII antibodies (Cedarlane Laboratories) for CAR1 expression in RIPA whole cell lysates. NK-GFP: Lentivirus-GFP stably transduced NK92MI/fCD16 cells. Data in (**D**) were presented as mean ± SEM.

After establishing these stable CAR1-NK cell lines, the expression of CAR1 and CD16 was verified in these cells by RT-PCR using primers for human fVII and CD3zeta for CAR1 and specific primers for CD16 and β actin (Fig. 2A–C) and by Sandwich ELISA using paired anti-human factor VII (hfVII) antibodies (Fig. 2D). In a published study, we showed that these anti-hfVII Abs in the ELISA assay kit could recognize fVII light chain in L-ICON1 protein^14^. Figure 2A showed that cDNAs of CAR1 monomer and dimer were detected in NK-CAR1 monomer and dimer cells, respectively, and their molecular masses (kilobase, kb) are consistent with their cDNA sequences (Supplementary Fig. S1**)**, whereas untransduced NK92MI cells were negative for CAR1. Figure 2B showed that CD16 cDNA was present in both NK-CAR1 monomer and dimer, but was not detected in the parental NK92MI control cells. Beta actin was used as a housekeeping gene control for equal amounts in RT-PCR assay (Fig. 2C). Paired fVII Ab ELISA (Fig. 2D) showed that CAR1 expression in the CAR1 dimer NK cells was two-fold (12.1 ng per μg RIPA lysates) of that in the CAR1 monomer NK cells (6.0 ng per μg RIPA lysates). The results of CAR1 expression was further verified and consistent (6.4 ng per μg dimer lysate and 3.6 ng per μg dimer lysate monomer lysates) (Supplementary Fig. S5a) when using M-PER, a different whole cell lysate buffer. Moreover, CAR1 was not detected in the supernatants of CAR-NK cell culture media (Supplementary Fig. S5b**)**, suggesting they are not secreted. Similarly, CAR1 expression was also detected on the cell surface on lentivirus-transduced 293 AD cells (Supplementary Fig. S5c**)**, further confirming that they are membrane-bound.

### TF-CAR-NK cells have direct killing effects against TNBC cells and also can mediate L-ICON ADCC to achieve a stronger effect

**Figure 3:**
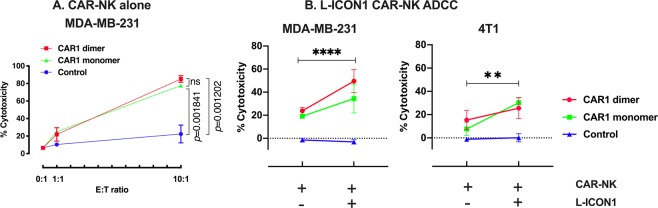
Cytotoxicity and ADCC effects (in combination with L-ICON1) of TF-CAR-NK cells *in vitro*. (**A**) Direct killing cytotoxicity of NK-CAR1 monomer and dimer (Effector cells) against human TNBC cell (Target cells, MDA-MB-231) was assayed using CytoTox Homogenous Assay Kit (Promega). Control cells were parental NK92MI cells. (**B**) Cytotoxicity of NK-CAR1 monomer, dimer (Effector cells) and NK92MI parental cell control to TNBC cells (Target cells, MDA-MB-231 and 4T1) was assayed in the presence or absence of L-ICON1 using CytoTox 96 non-radioactive cytotoxicity assay (Promega). Human IgG was used as isotype control in the control wells without L-ICON. Note that a suboptimal E:T ratio (1:1) in Panel B was used in order to show the enhanced effects when combined with L-ICON.

After having verified the expression of CAR1 and CD16 in NK-CAR1 monomer and dimer cells, I then tested and compared their ability (cytotoxicity) to kill human TNBC cells (MDA-MB-231) *in vitro*. The results in Fig. 3A showed that TF-CAR-NK cells exhibited significant direct cytotoxicity (mean ± SEM) against MDA-MB-231 cells (77.7 ± 2.2% with CAR1 monomer and 85.2 ± 3.8% with dimer) at an E:T ratio of 10:1 as compared to the control NK cells (monomer and dimer vs. control: *p* = 0.001841 and 0.001202, respectively), suggesting that TF-CAR-NK cells can kill TF-positive TNBC cells. The stronger cytotoxicity of CAR1 dimer (85.2%) than that (77.7%) of CAR1 monomer was possibly due to avidity of CAR constructs as CAR1 dimer expression was twice of CAR1 monomer on CAR1-NK cells (Fig. 2D and Supplementary Fig. S5a), however, the difference in cytotoxicity between CAR1 dimer and monomer was not statistically significant (*p* = 0.6786).

Next, I examined their ability to mediate L-ICON ADCC against human and murine TNBC cells (MDA-MB-231 and 4T1). We previously reported that TF expression was detected on both TNBC lines and hfVII light chain in L-ICON (hfVII light chain-IgG1Fc) could bind both human and murine TNBC lines^14^. The results in Fig. 3B showed that both TF-CAR-NK monomer and dimer cells exhibited stronger cytotoxicity against MDA-MB-231 (CAR1-NK monomer ADCC and dimer ADCC vs. control: 34.5 ± 12.4% and 49.6 ± 10.0%, *p* = 0.000987 and 0.000047, respectively) and 4T1 cells (monomer and dimer vs. control: 30.3 ± 4.6% and 25.6 ± 9.0%, *p* = 0.000045 and 0.001879, respectively) (E:T ratio = 1:1) in the presence of L-ICON1, as compared to those in the absence of L-ICON (with human IgG as an isotype control) (direct killing effect of monomer and dimer vs. control on MDA-MB-231: 19.2 ± 1.6% and 23.8 ± 3.0%, *p* = 0.000083 and 0.000193, respectively; monomer and dimer vs. control on 4T1: 7.7 ± 5.7% and 15.4 ± 8.3%, *p* = 0.037 and 0.008156, respectively), whereas the untransduced NK92MI control cells (CAR negative and CD16 negative) had no detectable killing effect either with or without L-ICON (Fig. 3B). We previously observed that ICON alone did not stop the proliferation of TF-positive cancer cells *in vitro* (through binding or blocking TF) in the absence of NK cells or complement^36^, which ruled out the possibility of direct killing of ICON and L-ICON through binding/blocking TF. Therefore, these results in Fig. 3B suggest that TF-CAR-NK cells can serve as effector cells to mediate L-ICON ADCC to achieve stronger effect than TF-CAR-NK cell alone.

Moreover, the killing activity of NK-CAR1 to MDA-MB-231 and 4T1 cells (Supplementary Videos 1 and 3) was observed in real-time and video recorded for a period of 3 days under IncuCyte Live-Cell imaging. In contrast, NK-GFP control cells could interact with TNBC cells, however, the cancer cells continued to proliferate in the assay plate wells (Supplementary Videos 2 and 4), suggesting that the control NK cells did not have killing activity to the cancer cells.

### TF-targeting CAR-NK cell therapy is effective and safe for the treatment of human TNBC in mouse models of orthotopic CDX and PDX

**Figure 4:**
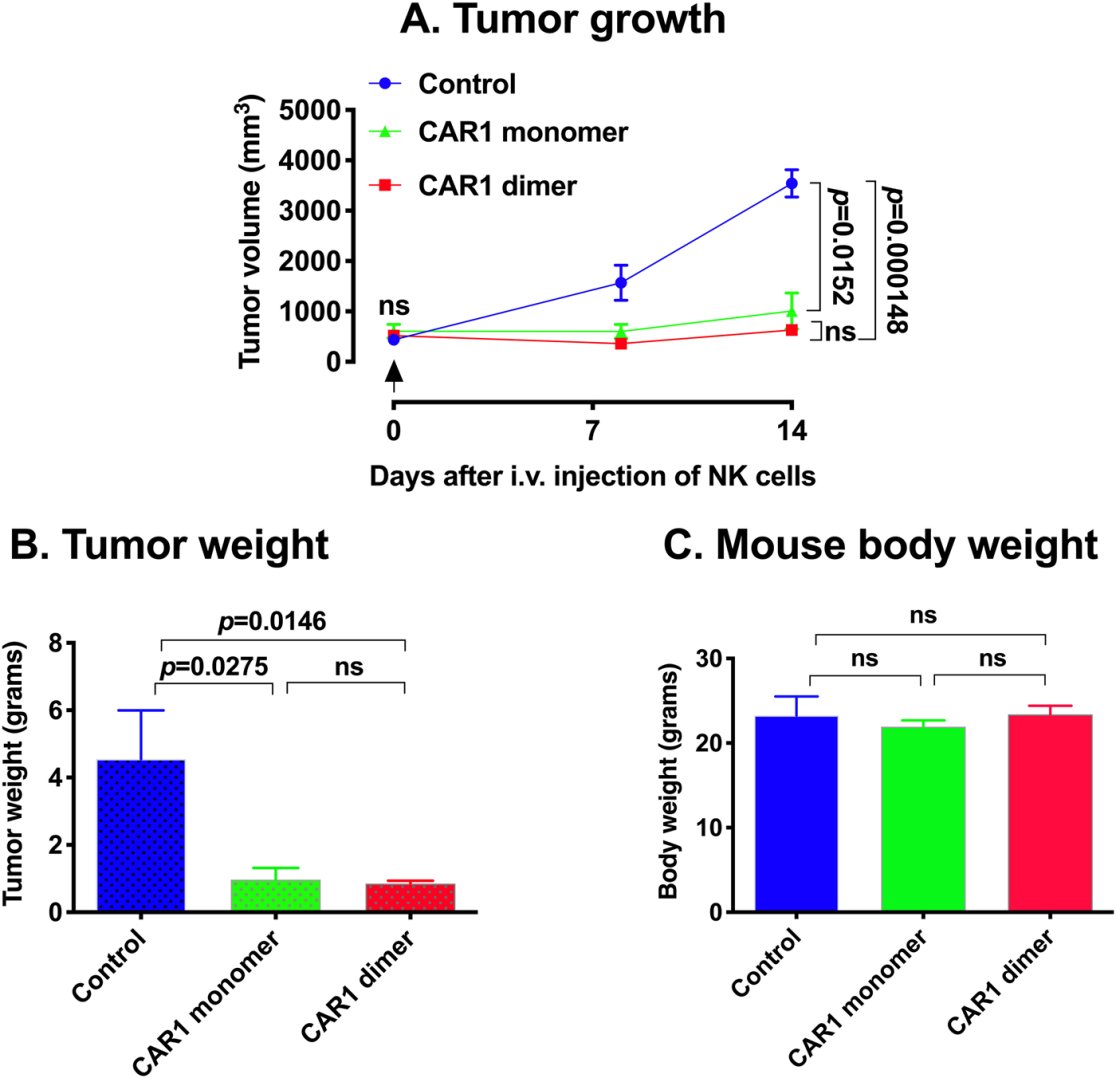
Efficacy and safety of TF-CAR-NK cells to TNBC cells *in vivo* for the treatment of TNBC CDX in an orthotopic NSG mouse model. Tumor volume (**A**), tumor weight (**B**) and mouse body weight (**C**) of female NSG mice that were treated with one i.v. injection of 2 × 10^6^ NK-CAR1 monomer, NK-CAR1 dimer or control NK92MI/fCD16 cells on day 0 (four mice per group). Data were analyzed by t test or ANOVA (Prism software) and were presented as mean ± SEM.

After having verified the *in vitro* effect of NK-CAR1 monomer and dimer cells, I then tested and compared their therapeutic efficacy and safety *in vivo* in an orthotopic TNBC CDX NSG mouse model. The results in Fig. 4 demonstrated that CAR1-NK monomer and dimer cells were effective and safe for the treatment of TNBC in an orthotopic CDX NSG mouse model. At the time when the mice were treated, the tumor xenografts had been well established with tumor volume (mean ± SEM) of 440 ± 99 mm^3^ in control group, 520 ± 44 mm^3^ in the NK-CAR1 dimer group and 605 ± 139 mm^3^ in the NK-CAR1 monomer group (four mice per group). After being treated once with i.v. injection of 2 × 10^6^ NK-CAR1 monomer or dimer cells, TNBC CDX growth was significantly arrested (71.5% and 82.1% reduction on day 14, *p* = 0.0152 and 0.000148, respectively, as compared to the control tumor volume). One control mouse died on day 0 right after i.v. injection of control cells. Another control mouse died on day 1 (~300 mm^3^ on day 0). The remaining control mice showed signs of sickness on day 14. One mouse treated with NK-CAR1 monomer died on day 14, which was more likely due to the tumor burden (865 mm^3^ on day 8) and was unlikely due to the NK-CAR1 treatment, as all other NK-CAR1 dimer and monomer treated mice appeared healthy at the time of sacrifice on day 14. Similar to the *in vitro* data, NK-CAR1 dimer cells were slightly more effective for treating TNBC CDX than NK-CAR1 monomer, as evidenced by measuring tumor volume (82.1% vs. 71.5% reduction on day 14, *p* = 0.2846, as compared to that of control tumors) (Fig. 4A) and tumor weights (0.850 ± 0.087 grams, n = 4, vs. 0.967 ± 0.348 grams, n = 3, *p* = 0.7210, as compared to control tumor weight 4.550 ± 1.450 grams, n = 2) (Fig. 4B), however, the difference between CAR1 dimer and monomer was not statistically significant (*p* > 0.05 for tumor volume and tumor weight).

Body weight was assessed as an indicator of safety, per previously recorded models^14,29,45^. There was no statistical difference in net mouse body weights between control and NK-CAR1 treated groups (all *p* > 0.05) (Fig. 4C), suggesting that TF-targeted CAR1-NK therapy was safe. Since TF-CAR1 dimer cells exhibited stronger effects than TF-CAR1 monomer cells for the treatment of TNBC *in vitro* (Fig. 3) and *in vivo* (Fig. 4), NK-CAR1 dimer, but not monomer, was used in the subsequent studies.

Patient-derived xenograft (PDX) is considered a better animal model than CDX to predict clinical outcome. To better translate TF-CAR-NK therapy into the clinic, therefore, we tested the efficacy and safety of NK-CAR1 cell therapy in a pilot study in an orthotopic SCID mouse model of TNBC PDX (with BRCA1 mutation)^14^. Supplementary Fig. S6 demonstrated that TF-CAR1 dimer cell immunotherapy was effective and safe for the treatment of TNBC PDX in the orthotopic mouse model, as determined by measuring tumor volume (87.9% reduction as compared to the control tumor on day 31 after initiation of treatment, *p* = 0.0043) (Supplementary Fig. S6a), tumor weight (3.6 ± 0.9 grams of control tumor vs. 0.8 ± 0.3 grams of CAR1 dimer-treated tumor on day 31, *p* = 0.0235) (Supplementary Fig. S6b) and net mouse body weight (average 19.0 grams of control mice vs. 21.1 grams of CAR1 dimer-treated mice on day 31, *p* = 0.1013) (Supplementary Fig. S6c).

**Figure 5:**
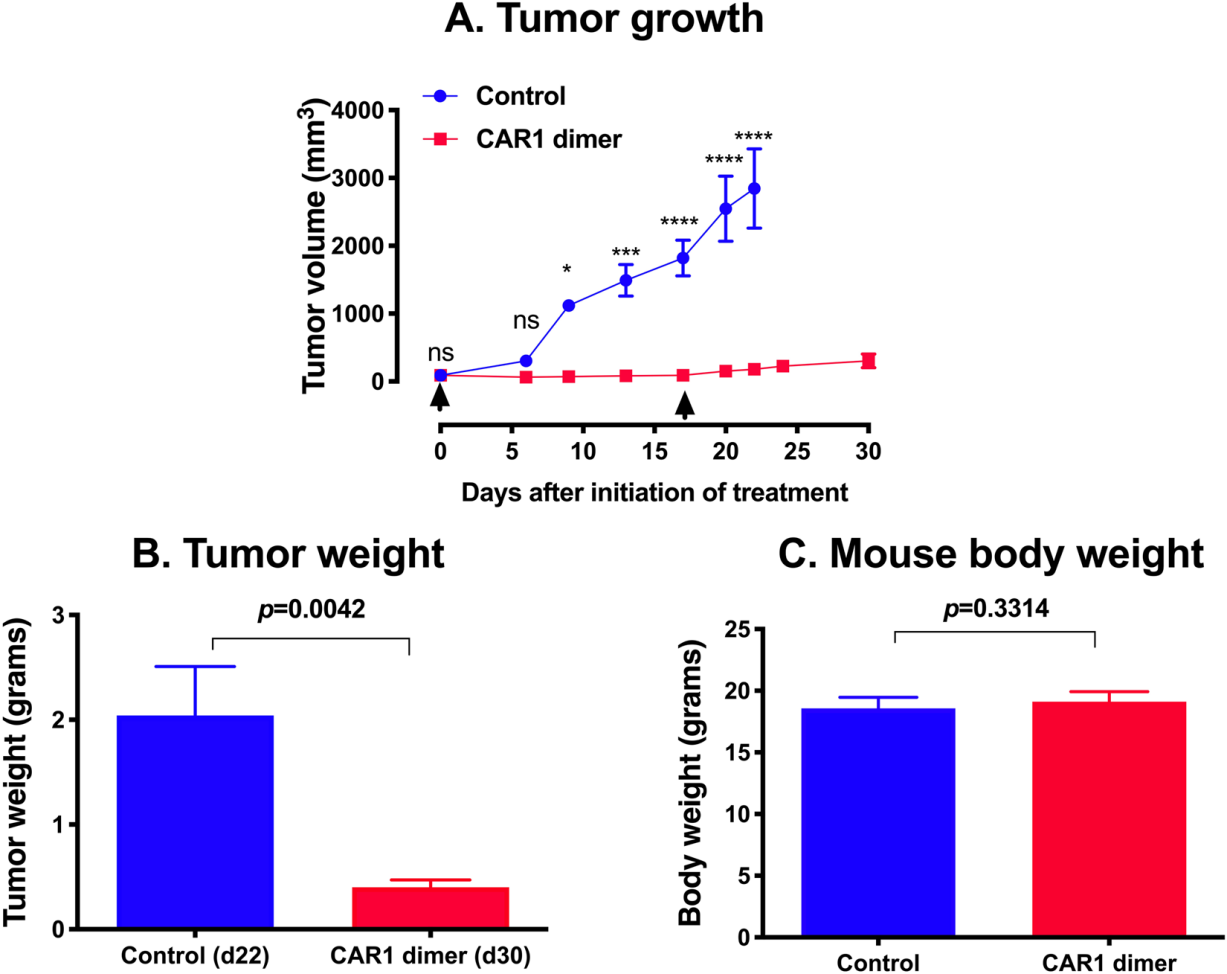
TF-CAR-NK cell therapy was effective and safe for the treatment of human TNBC PDX in an orthotopic NSG mouse model. TNBC PDX (JAX) was generated in four-week old female NSG mice, as described above and in our published study^14^. When the PDX tumors reached ~100 mm^3^, NK-CAR1 dimer (NK92MI/fCD16/CAR1 dimer) or NK92MI/fCD16 cells without CAR constructs (as a control) were injected i.v. via tail veins two times (3 × 10^6^ cells per mouse on day 0 and 2 × 10^6^ cells per mouse on day 17, arrows) (five mice per group). (**A**) Tumor volumes. (**B**,**C**) Tumor weights and mouse body weights were measured at the time of sacrifice of animals. ns: no significance; *, **, ***, ****p < 0.05, 0.01, 0.001 and 0.0001.

To verify the findings observed in the pilot study, we repeated the efficacy and safety study of TF-CAR1 dimer therapy one more time in the orthotopic TNBC PDX model in NSG mice. The results demonstrated that TF-targeting CAR1-NK cells inhibited orthotopic PDX growth in mice as measure by tumor volume (*p* < 0.0001, 93.7% reduction as compared to control tumor on day 22 after initiation of treatment) (Fig. 5A). Control mice had to be euthanized on day 22 due to their tumor burden. Tumor weights also showed a significant difference *ex vivo* (*p* = 0.0042, NK-CAR1 dimer treated tumors on day 30 vs. control tumors on day 22) (Fig. 5B), whereas net mouse body weights had no difference between control mice and treated mice (*p* = 0.3314, for 18.6 grams of control mice vs. 19.1 grams of treated mice) (Fig. 5C).

## Discussion

An important goal for the development of new targeted cancer immunotherapy will be the identification of new surface targets that, ideally, are common yet specific to most or all major and important tumor compartments in the tumor microenvironment, notably the cancer cells, CSCs and tumor VECs, whereas their expression on normal cells is minimal or is restricted to the normal cells that are sequestered from systemically administered therapeutic agents. Accumulating evidence suggest that TF is a useful surface target in patients with TNBC and non-TNBC breast cancer, and more general, in solid cancers. On one end, in the tumor microenvironment, TF expression is highly detected on the cancer cells in cancer patients with a variety of solid cancer^23^, including both TNBC and non-TNBC, for example, 50–85% of patients with TNBC^14^ and 81–100% of patients with non-TNBC^16,46,47^. TF was also specifically expressed on tumor VECs in tumors from patients with TNBC^14^ and non-TNBC breast cancer^16^ as well as in human chemoresistant non-TNBC and TNBC tumor xenografts^14,45^ from mice, whereas TF is not expressed on normal VECs in adjacent breast tissues^14,16^. Thirdly, we demonstrated that TF was also expressed on breast cancer stem cells (CD133+ and CD24-CD44+) isolated from human breast cancer cell lines, tumor xenografts and patients’ breast tumors^15^.

On the other end, in normal physiological conditions, TF is not expressed on normal leukocytes in circulation^23^, such as B, T, NK cells and monocytes, and not on quiescent VECs of normal blood vessels in normal organs^22,23,29,48,49^. TF occurs only in cells that are not in direct contact with the blood, such as pericytes, which are localized in the sub-endothelial vessel wall, and the epithelial cells of the skin, mucosa and glomeruli^22,23^. Thus, under normal conditions, TF expression is sequestered from its natural ligand^17,19,50^, fVII^22,23^, in circulation. Upon disruption of vessel wall integrity, TF in pericytes and smooth muscle cells is released and can be bound by fVII, leaking from blood circulation, to initiate coagulation^20,51^. In addition to its role as the primary initiator of normal coagulation, TF is also a modulator of pathological angiogenesis in tumor, endometriosis and AMD^24–26,52–54^. TF expression on angiogenic VECs is well documented in the pathological neovasculature of cancer^16,24,28,29,35,45^, AMD^55^ and endometriosis^56^ from animal models to patients. However, TF is not expressed on normal VECs. Moreover, an important finding made by an independent group demonstrated that TF is not expressed on the angiogenic VECs in physiological angiogenesis such as skin wound healing^22,57^. Thus, the findings of high expression rate of TF in cancer yet minimal/restricted expression in normal cells make TF an ideal and promising target for development of targeted immunotherapy.

To target TF for antibody immunotherapy, Hu and Garen previously developed the first TF-targeting Immunoconjugate agent ICON^27,28,35^. ICON therapy of preclinical, experimental murine and human solid cancers, including melanoma^27^, prostate cancer^35^, and head and neck cancer^37^ leads to marked tumor growth inhibition^27,28^ or complete eradication^35,37^ of well-established tumor xenografts and distant metastases^27,35^ with no detectable side effects on normal tissues^27,56^. As an IgG1 therapeutic antibody, ICON efficacy is mediated by NK cells via ADCC and by complement-dependent cytotoxicity (CDC)^37^. In particular, NK activity and levels are crucial for *in vivo* ICON efficacy^37^. As a neovascular-targeting agent, ICON has shown efficacy and safety, not only for the treatment of cancer, but also for other pathological angiogenesis-dependent human diseases, notably wet age-related macular degeneration (AMD)^55,58^ and endometriosis^56,59^ in preclinical animal (mouse, rat, pig and non-human primate) models and in early phase clinical trials. ICON was well tolerated and already showed biological activity with sub-optimal therapeutic dosages in completed Phase I and II trials in AMD patients (Clinical trial identifier # NCT01485588 and NCT02358889). Moreover, ICON is being tested in a Phase I trial for ocular melanoma (NCT02771340). Our lab has recently improved ICON to a second-generation ICON (GenBank accession KX760097) with improved efficacy and safety profiles, named L-ICON or L-ICON1 for lighter or light chain ICON^14^. As compared to ICON, L-ICON1 consists of only the light chain (the first 152 aa) of fVII fused to an IgG1 Fc, making it >50% smaller MW and complete depletion of pro-coagulation activity with intact binding activity to cancer cells. It is more effective than the original ICON *in vivo* for the treatment of TNBC in mouse models of orthotopic CDX as well as effective and safe in the TNBC PDX model^14^. The study also demonstrated that light chain of human fVII in L-ICON1 can bind to TF on both murine and human cancer cells better than mouse fVII light chain in mouse L-ICON1 and hfVIIK341A in human ICON, as determined by flow cytometry and ELISA^14^.

To target TF for cellular immunotherapy, I describe in this study, to my knowledge for the first time, the development and testing of TF-targeted human CAR constructs, which are composed of human fVII light chain, with or without a hinge region of human IgG1, followed sequentially by CD28 transmembrane and cytoplasmic domains and then by the cytoplasmic domains of 4-1BB and CD3ζ (TF-CAR1 monomer and dimer) (Supplementary Fig. S1). In the current TF-CAR1 constructs, I made a monomer and a dimer to test whether the effects of TF-CAR-NK cells would be stronger in dimer than monomer through TF binding (avidity) and/or dimerization of CAR receptors. NK-CAR1 dimer cells indeed exhibited slightly, but not statistically significantly, stronger effects than NK-CAR1 monomer cells in *in vitro* and *in vivo* assays. In addition, I designed two unique restriction enzyme sites at the 5′- and 3′-end of 4-1BB (Supplementary Fig. S1) so that 4-1BB can be replaced with OX40 in our future study to generate another third generation TF-CAR (as TF-targeting CAR2) or OX40 can be inserted in frame between 4-1BB and CD3ζ as a fourth generation TF-CAR (named TF-targeting CAR3).

Similar to our previous idea of the use of murine fVII single chain peptide^27,28,35^ and light chain^14^ to target murine and human TF, a published study used murine fVII light chain in construction of TF-targeting CAR T cells (CD8 hinge and transmembrane followed by CD28, 4-1BB and CD3ζ)^60^. In the design and development of TF-targeting CAR, I chose human fVII light chain, the natural ligand for TF, instead of full length human fVII, partial sequence of murine fVII, full length antibody or antibody fragments (e.g., scFv) against TF, as the recognition domain for TF for the following reasons. First, the affinity of fVII for TF^61^ is about 100- to 1000-fold greater than antibody for TF antigen^62^. Second, fVII can be synthesized in human origin by recombinant DNA technology. Furthermore, our unexpected results showed that the light chain of human fVII bound significantly better than full length human fVII and murine fVII light chain to TF-expressing human and murine cancer cells^14^. In addition, the TF-CAR1 NK cells that we constructed also express CD16 so that they can serve as effector cells to potentially mediate ADCC in combination therapy with ICON^37^, L-ICON^14^ and other therapeutic antibodies for cancer immunotherapy. NK92 and NK92MI cells have been used in preclinical and clinical studies^63,64^. The results showed that NK92MI-based CAR1-NK cells were effective and safe for the treatment of TNBC *in vitro* and *in vivo* in mouse models and that they could mediate L-ICON ADCC to achieve additional killing effects on TF-expressing cancer cells *in vitro*. However, NK92-derived CAR-NK cells have a number of disadvantages in clinical applications^63^. For example, they must be irradiated before infusion into patients but irradiation may reduce their *in vivo* proliferation, persistence or killing activity^41,63^. To better translate the finding of this study in patients with TNBC, NK cells from cord blood, and embryonic/pluripotent stem cells, patients’ autologous NK cells or *ex vivo* expanded NK cells^65,66^ will be used to generate clinical grade TF-CAR-NK cells. Combination therapy of TF-CAR-NK cells and L-ICON (or other therapeutic antibody) will be further studies *in vivo* in animal models of cancer when using TF-CAR-NK cells generated from healthy donors’ and cancer patients’ NK cells.

Lentiviruses can infect both dividing and non-dividing cells and are being used to transduce a wide range of cell types, including NK and T cells. In addition to transducing NK92MI cells, this study also demonstrated that the lentivirus TF-CAR constructs could transduce Jurkat T cell line as a T cell model (Supplementary Fig. S3). Therefore, the TF-CAR constructs are also being explored in the lab for the development of TF-CAR-T cells. The lentivirus transduction efficiency in this study was relatively low, probably due to the fact that the lentiviral titer in this study was not determined so that the MOI used might not have been optimal. Nevertheless, TF-CAR-NK cells had been stably selected with puromycin and had been verified for CAR1 and CD16 expression before they were used in all *in vitro* and *in vivo* studies. In our follow-up studies, in addition to lentivirus transduction, we may also test non-viral transduction techniques such as transposon or mRNA electroporation to generate TF-CAR cells using healthy donors’ and/or patients’ NK and T cells.

In summary, accumulating preclinical and clinical evidence suggests that TF is a useful target for cancer therapy because of its common yet selective expression on several important tumor compartments and its minimal or restricted expression in normal cells. Several TF-targeting therapeutics, including ICON^35^, L-ICON^14^ and CAR-NK (and -T) cells therapies and antibody-drug conjugates (ADC)^67,68^, are under preclinical and clinical evaluation. In particular, this study established the proof of concept of targeting TF as a new target for CAR-NK cellular immunotherapy for the treatment of TNBC and may warrant further investigation in TNBC patients. If the efficacy and safety can be proven in clinical trials, TF-targeted therapeutics may represent a novel therapeutic approach for TNBC and can potentially impact the treatment regimen for pathological angiogenesis-dependent human diseases (notably cancer, age-related macular degeneration, endometriosis and rheumatoid arthritis) and macrophage-associated human disease (such as atherosclerosis, HIV and Ebola viral transduction)^23^, in which TF is selectively expressed on angiogenic VECs and/or disease-associated macrophages^23^.

## Methods

### Cell lines

The cell lines were used for producing lentivirus (293TN), testing lentiviral transduction (NK92MI, ADCC effector cells, 293AD) and CAR expression (NK92MI), generating CAR-NK cells (NK92MI), or testing CAR-NK cytotoxicity (TNBC cell lines MDA-MB-231 and 4T1). NK92MI cells were purchased from American Type Culture Collection (ATCC) in 2007 and were grown in Alpha Minimum Essential medium (MEMα) without ribonucleosides and deoxyribonucleosides (ATCC). Prior to lentivirus transduction, I have transfected NK92MI with plasmid pcDNA3.1(+) encoding full length human CD16 (fCD16) (with G418 resistance) to generate stable NK92MI/fCD16 cells under selection of G418 (500 μg/ml and then 100 μg/ml for passaging and maintaining the cells). 293TN (System Biosciences, SBI) and 293AD (Cell Biolabs) were purchased from commercial vendors in 2017 and 2014, respectively, and were grown in DMEM medium (high glucose) supplemented with 10% heat inactivated FBS (HI-FBS) (to inactivate complement), 0.1 mM MEM Non-Essential Amino Acids, 2 mM L-glutamine and 1 × penicillin and streptomycin (Invitrogen). ADCC effector cells, a Jurkat-based T cell line transfected with CD16 and N-FAT/Luciferase, were purchased from Promega in 2014 and were grown in RPMI 1640 complete growth medium supplemented with 100 μg/ml G418, 250 μg/ml hygromycin, 10% HI-FBS, 1 × nonessential amino acids, 1 × sodium pyruvate and 1 × penicillin and streptomycin (Invitrogen). Human TNBC cell line MDA-MB-231 (BRCA1-wt, BRCA2-mutant) were purchased from ATCC in 2009 and were grown in L-15 medium. Murine TNBC 4T1 cells were grown in RPMI1640 medium^14^. All media were supplemented with 10% heat-inactivated fetal bovine serum (HI-FBS) (Sigma) and 1 × penicillin and streptomycin (Invitrogen) except that MEMα for NK92MI cells were supplemented with 0.1 mM 2-mercaptoethanol (Invitrogen), 12.5% horse serum (Sigma) and 12.5% HI-FBS (Sigma). Cultures were tested annually for mycoplasma contamination using Mycoalert Mycoplasma Detection Kit (Lonza). Authenticity of cancer cell lines was determined by validating TF expression by flow cytometry, Western blots and/or cell ELISA, as described^14^.

### Construction of CAR plasmid DNAs and lentiviral vectors

The TF-targeting CAR1 dimer and monomer constructs that I designed in this study encode human factor VII light chain (as TF recognition domain) with human IgG1 hinge region (to form a homodimer of CAR1) or without the hinge region (as monomer CAR1) followed by CD28 transmembrane and cytoplasmic domains, 4-1BB and CD3 zeta. The CAR1 cDNA constructs were custom synthesized by SBI into the multi-cloning sites of lentiviral shuttle vector pCDH (SBI Cat. No. CD713B), which also encodes green fluorescence protein (GFP). The cDNA sequences for TF-targeting CAR1 monomer and dimer were verified by Sanger DNA sequencing with sequencing primers (5′-primer for MSCV and 3′-primer for EF1, SBI) for pCDH vector at SBI before shipping to my laboratory and were verified again at The Ohio State University Sanger DNA Sequencing Core after I received the plasmid DNA constructs. The cDNA sequences of TF-targeting CAR1 are deposited at GenBank with accession numbers MF806378 and MF806379, respectively. The lentiviruses encoding CAR1 dimer and monomer were produced by co-transfecting 293TN (SBI) with pCDH-CAR1 and pPACKH1 (SBI) using PurFection transfection reagent (SBI) following the manufacturer’s instructions. The control lentivirus, lenti-GFP, was produced by co-transfecting 293TN cells with the backbone pCDH encoding GFP only (SBI, Cat. No. CD713B) with pPACKH1 (SBI). Thus, the only difference between Lenti-CAR1 and Lenti-GFP is that the latter does not encode a CAR construct. The lentivirus in cell culture supernatant was collected on 48 and 72 hours and used as unconcentrated viral particles in supernatant (SNT) or concentrated using PEG-it reagent (SBI) following the manufacturer’s instruction.

### Stable transduction and expression of CAR1 dimer, monomer or control GFP on adherent (293AD) and suspension cells (NK92MI and ADCC effector cells)

The cells were transduced with lentivirus in the presence of TransDUX and MAX Enhancer (SBI) following the manufacturer’s instructions. Briefly, 50,000 cells per well of either adherent or suspension cells were seeded in a 24 well plate in cell culture medium. The next day, the cell culture medium was replaced with 500 μl transduction medium, containing 2.5 μl of TransDux (SBI), 100 μl of MAX Enhancer and 400 μl culture medium. Then 150 μl of 293TN lentivirus-containing supernatant (SNT) was added to each well, and plates were swirled to mix. By 72 hours (hrs) post-transduction, the viral genome has integrated into the host cell genome and the cells were transferred and grown in complete growth medium supplemented with 5 μg/ml puromycin for 293AD, with 5 μg/ml puromycin and 100 μg/ml G418 for NK-GFP and NK-CAR1 monomer and dimer cells or with 5 μg/ml puromycin, 100 μg/ml G418 and 250 μg/ml hygromycin for ADCC effector cells to establish stable cell lines. After untransduced control cells were killed under the same selections of respective antibiotics, lenti-CAR- or lenti-GFP-transduced stable cell lines were expanded and then frozen for long-term storage in liquid nitrogen freezer. For the cells used in experiments below, they were grown and maintained in complete growth medium supplemented with 1 μg/ml vitamin K1 (Sigma) and antibiotics at their respective concentrations above.

### Expression of CAR1 on CAR1-NK cells by RT-PCR and ELISA

RT-PCR and ELISA were carried out to verify expression of CAR1 and CD16 on lentivirus-transduced or plasmid-transfected cells. Total RNAs were extracted from NK92MI CAR1 dimer, monomer and untransduced cells using TriZol Reagent (Invitrogen). RT-PCR for CAR1, CD16 and beta actin was performed using One-Step RT-PCR kit (New England Biolabs) with corresponding primers (Supplementary Table S1) and was analyzed by agarose gel electrophoresis. RIPA lysis buffer (Santa Cruz) and M-PER Extraction buffer (Thermo Fisher) were used to extract whole cell lysates from NK92MI untransduced and lenti-transduced CAR1 dimer, monomer and GFP only cells. Protease inhibitors (Santa Cruz and Sigma) were added to RIPA (Santa Cruz) and M-PER (Thermo Scientific) lysis buffers, respectively, prior to use. Protein concentrations in the cell lysates were determined by Protein Assay Reagent (Bio-Rad) using Bovine Serum Albumin standard protein (Pierce). CAR1 concentration was assayed by Paired Human Factor VII Antibody Sandwich ELISA (Cedarlane Laboratories) using recombinant L-ICON1 protein as standards following the published procedure with a minor modification (1:200 dilution for capture and detection Abs)^14^. CAR1 expression was normalized and presented as ng per μg cell lysate.

### Direct cytotoxicity and ADCC (with L-ICON) of CAR1-NK cells against TNBC cells

Direct cytotoxicity of CAR1-NK cells was determined using LDH release-based CytoTox-One homogenous membrane integrity assay (Promega) following the manufacturer’s instruction. Briefly, TNBC cells (MDA-MB-231 and 4T1, target cells) were seeded 10,000 cells per well in 100 μl growth medium supplemented with 10% heat-inactivated FBS in 96-well U-bottom Tissue Culture-treated microplates overnight. The next morning, NK92MI/fCD16 expressing CAR1-dimer, -monomer (as effector cells), untransduced NK92MI or NK92MI/Lenti-GFP cells (as CAR-negative control cells) were resuspended in DMEM with 0.5% super low IgG HI-FBS and added to the wells at E:T ratio of 10:1, 1:1 and 0:1. After 4 hours incubation at 5% CO_2_ and 37 °C, the microplate was centrifuged at 300 × g for 5 min and 50 μl of supernatant was transferred to a new 96-well flat-bottom microplate. Cytotoxicity assay solution was added (50 μl per well) and incubated at 37 °C for 30 min before stop solution was added following the manufacturer’s instructions. For CytoTox One homogeneous assay, fluorescence at Ex560 nm and Em590 nm was read on a microplate reader (Molecular Devices, i3). Cytotoxicity percent was calculated using the formulas described in the manufacturer’s instructions.

CAR1-NK-mediated L-ICON ADCC effects were determined using CytoTox 96 non-radioactive cytotoxicity assay (Promega) following the manufacturer’s instruction. The procedure was similar to cytotoxicity assay above except that L-ICON1 protein was added (to a final concentration of 10 μg/ml) and incubated with TNBC cells at 37 °C for 30 min prior to the addition of CAR1-NK or control NK cells. In the control wells, L-ICON1 was replaced by human IgG (10 μg/ml; Sigma) as isotype control followed by 4-hour incubation with CAR1-NK or control NK cells. In the ADCC assay, a suboptimal E:T ratio (1:1) was used in order to observe the effects through L-ICON-ADCC. After the 4-hour incubation, 50 μl of supernatant was transferred to a new 96-well microplate and 50 μl of CytoTox 96 Reagent was added and incubated for 30 minutes at room temperature and then stopped by adding 50 μl Stop Solution. Absorbance at 490 nm was recorded (Molecular Devices, i3) and cytotoxicity percent was calculated using the formulas described in the manufacturer’s instructions.

The cytotoxicity of TF-CAR-NK cells against TNBC cells (MDA-MB-231 and 4T1) were further observed in real-time under Live-Cell IncuCyte Analyses (sartorius). Briefly, TNBC cells (1000 cells per well) were seeded in triplicate in inner wells of a 96 well cell culture microplate and grown in DMEM supplemented with 2.5% super low IgG FBS at 5% CO_2_ and 37 °C overnight, as similarly described in ADCC Effector Assay^14^. Next morning, TF-CAR-NK cells (CAR1 dimer and monomer) and GFP-NK control cells were added at a ratio of 10:1. The microplate was placed in Live-Cell IncuCyte incubate (sartorius) and cytotoxicity of TF-CAR-NK cells against the cancer cells in each well was recorded every 2 hours for a period of 3 days. The results were exported as MPEG-4 movie files and provided as Supplementary Materials.

### Generation of orthotopic mouse models of TNBC cell-derived xenografts (CDX) and patient-derived xenografts (PDX)

The animal study protocol was reviewed and approved by the Institutional Animal Care and Use Committee of The Ohio State University (OSU IACUC #2013A00000047-R2). All experiments were performed in accordance with relevant guidelines and regulations. The procedures have been described in details in a published study^14^. Briefly, to generate orthotopic mouse models of tumor line-derived xenografts, 5 × 10^5^ human TNBC MDA-MB-231/Luc + GFP cells in 50 μl of PBS were injected into the fourth left mammary gland fat pad in 4–6 weeks-old, female NSG (Taconic Farms). To generate an orthotopic TNBC PDX model, we purchased a TNBC PDX donor NSG mouse **(**NOD SCID gamma, absent mature B, T, NK cells and absent complement**)** with BRCA1 mutation from Jackson Laboratory (JAX TM00089, breast tumor markers: TNBC ER-/PR-/HER2-, BRCA1 V757fs). Surgeries were performed in accordance with the OSU IACUC Rodent Surgery Policy. Mice were anesthetized with isoflurane. For the donor NSG mouse, an incision was made over the flank of tumor to extract the tumor tissue, which was washed once in RPMI medium supplemented with 1 x penicillin and streptomycin and was cut in sterile PBS into ~3 millimeters (mm) pieces in diameter for implantation into the recipient mice. NSG mice (and CB-17 SCID mice in the pilot study) (Taconic Farms) were used as recipient mice. For recipient mice, an incision was made over the fourth right mammary gland, for direct implantation of one piece of ~3 mm PDX tumor tissues from the donor tumor tissue into it. One drop of tissue adhesive (Vetbond) and/or 1 wound clip (Sigma) was used to close the incision site in routine fashion. Animals were monitored for recovery from anesthesia before returning to routine husbandry. Animals were checked daily until wounds were healed, and analgesics (buprenorphine, s.c. 0.1 mg/kg in 100 μl sterile PBS) were administered to provide pain relief for up to 72 hours after surgery.

### *In vivo* studies of TF-CAR-NK cell therapy

TNBC CDX (MDA-MB-231/Luc + GFP) and PDX (JAX TM00089) were generated in four-week old female NSG mice, as described above as well as in details in our published study^14^. When the orthotopic tumors were well established (CDX at an average tumor volume of ~500 mm^3^ and PDX at ~100 mm^3^), NK92MI expressing CD16 and CAR1 dimer or monomer (CAR1 dimer and monomer) were injected intravenously (i.v.) once or twice every two weeks via tail veins (2 to 5 × 10^6^ cells per mouse). Control mice were i.v. injected with untransduced NK92MI/fCD16 cells. Therapeutic efficacy was determined by measuring tumor width (W) and length (L) with calipers in millimeters (mm) and calculating tumor volume (mm^3^) using the formula (W × W × L)/2 (mm^3^), as described^35,37^. At the time of sacrifice of the animal, whole mouse body weights (grams) were measured before tumor xenograft was removed and then tumor xenografts were removed and tumor weights (grams) were measured. Net body weights (grams) were actually measured (after removal of tumor) or by subtracting tumor weights from the whole mouse body weights.

### Statistical analysis

The data *in vitro* and *in vivo* are presented as mean ± SEM and analyzed by one-way or two-way ANOVA and t-test for statistical significance using Prism software (GraphPad). For analyses of statistical significance, duplicate or triplicate wells in each group were used for *in vitro* assays in tissue culture plates and 5 mice per group were used for *in vivo* studies in animal (unless specified). Statistical significance is presented as *P < 0.05; **P < *0.01*; ***P < 0.001; ****P < *0.0001* and “ns” stands for no (statistical) significance.

### Accession codes

The cDNA sequences of TF-targeted CAR1 monomer and dimer generated during the current study are available in the GenBank with accession no. MF806378 and MF806379. The material may be available for research via Material Transfer Agreement through The Ohio State University Office of Technology and Commercialization Office.

## Supplementary information

Supplementary Information.

Video 1.

Video 2.

Video 3.

Video 4.
