# Supplementary material for: Tissue factor as a new target for CAR-NK cell immunotherapy of triple-negative breast cancer
Source: Sci Rep. 2020 Feb 18;10:2815. doi: 10.1038/s41598-020-59736-3 (PMC7028910; doi:10.1038/s41598-020-59736-3)
Supplement: Supplementary file 1 — Supplementary Information. [file 41598_2020_59736_MOESM1_ESM.pdf]

Supplementary Material

Tissue factor as a new target for CAR-NK cell immunotherapy of triple-negative breast cancer

Zhiwei Hu*

Department of Surgery, Division of Surgical Oncology, The Ohio State University Wexner Medical Center and The OSU James Comprehensive Cancer Center, Columbus, OH 43210, USA

*Zhiwei.hu@osumc.edu

Supplementary Figures: 9

Supplementary Table: 1

Supplementary videos: 4

A. Tissue factor-targeting CAR1 monomer (1300 bp)

GCTAGCGCCACC**ATG**GTCTCCCAGGCCCTCAGGCTCCTCTGCCTTCTGCTTGGGCTTCAGGGCTGCCTGGCTGCAGT
CTTCGTAACCCAGGAGGAAGCCCACGGCGTCCTGCACCGGCGCCGGCGCGCCAACGCGTTCCTGGAGGAGCTGCGGC
CGGGCTCCCTGGAGAGGGAGTGCAAGGAGGAGCAGTGCTCCTTCGAGGAGGCCCGGGAGATCTTCAAGGACGCGGAG
AGGACGAAGCTGTTCTGGATTTCTTACAGTGATGGTGACCAGTGTGCCTCAAGTCCATGCCAGAATGGGGGCTCCTG
CAAGGACCAGCTCCAGTCCTATATCTGCTTCTGCCTCCCTGCCTTCGAGGGCCGGAACTGTGAGACGCACAAGGATG
ACCAGCTGATCTGTGTGAACGAGAACGGCGGCTGTGAGCAGTACTGCAGTGACCACACGGGCACCAAGCGCTCCTGT
CGGTGCCACGAGGGGTACTCTCTGCTGGCAGACGGGGTGTCCTGCACACCCACAGTTGAATATCCATGTGGAAAAAT
ACCTATTCTAGAAAAAAGAAATGCCAGCAAGCCCCAAGGGCGAGGATCCTTTTGGGTGCTGGTGGTGGTTGGTGGAG
TCCTGGCTTGCTATAGCTTGCTAGTAACAGTGGCCTTTATTATTTTCTGGGTGAGGAGTAAGAGGAGCAGGCTCCTG
CACAGTGACTACATGAACATGACTCCCCGCCGCCCCGGGCCCACCCGCAAGCATTACCAGCCCTATGCCCCACCACG
CGACTTCGCAGCCTATCGCTCCTTCGAACGTTTCTCTGTTGTTAAACGGGGCAGAAAGAAACTCCTGTATATATTCA
AACAACCATTTATGAGACCAGTACAAACTACTCAAGAGGAAGATGGCTGTAGCTGCCGATTTCCAGAAGAAGAAGAA
GGAGGATGTGAACTGGAATTCAGAGTGAAGTTCAGCAGGAGCGCAGACGCCCCCGCGTACCAGCAGGGCCAGAACCA
GCTCTATAACGAGCTCAATCTAGGACGAAGAGAGGAGTACGATGTTTTGGACAAGAGACGTGGCCGGGACCCTGAGA
TGGGGGGAAAGCCGAGAAGGAAGAACCCTCAGGAAGGCCTGTACAATGAACTGCAGAAAGATAAGATGGCGGAGGCC
TACAGTGAGATTGGGATGAAAGGCGAGCGCCGGAGGGGCAAGGGGCACGATGGCCTTTACCAGGGTCTCAGTACAGC
CACCAAGGACACCTACGACGCCCTTCACATGCAGGCCCTGCCCCCTCGC**TGA**GTTTAAACGCGGCCGC

NheI-Kozak-fVII light chain (Start codon + signal + mature fVII light chain)-BamHI-CD28 (TM+CD)-BstBI-4-1BB-EcoRI-CD3zeta CD-Stop codon-PmeI-NotI

B. Tissue factor-targeting CAR1 dimer (1354 bp)

GCTAGCGCCACC**ATG**GTCTCCCAGGCCCTCAGGCTCCTCTGCCTTCTGCTTGGGCTTCAGGGCTGCCTGGCTGCAGT
CTTCGTAACCCAGGAGGAAGCCCACGGCGTCCTGCACCGGCGCCGGCGCGCCAACGCGTTCCTGGAGGAGCTGCGGC
CGGGCTCCCTGGAGAGGGAGTGCAAGGAGGAGCAGTGCTCCTTCGAGGAGGCCCGGGAGATCTTCAAGGACGCGGAG
AGGACGAAGCTGTTCTGGATTTCTTACAGTGATGGTGACCAGTGTGCCTCAAGTCCATGCCAGAATGGGGGCTCCTG
CAAGGACCAGCTCCAGTCCTATATCTGCTTCTGCCTCCCTGCCTTCGAGGGCCGGAACTGTGAGACGCACAAGGATG
ACCAGCTGATCTGTGTGAACGAGAACGGCGGCTGTGAGCAGTACTGCAGTGACCACACGGGCACCAAGCGCTCCTGT
CGGTGCCACGAGGGGTACTCTCTGCTGGCAGACGGGGTGTCCTGCACACCCACAGTTGAATATCCATGTGGAAAAAT
ACCTATTCTAGAAAAAAGAAATGCCAGCAAGCCCCAAGGGCGAGGATCCGCAGAGCCCAAATCTTGTGACAAAACTC
ACACATGCCCACCGTGCCCAGGATCCTTTTGGGTGCTGGTGGTGGTTGGTGGAGTCCTGGCTTGCTATAGCTTGCTA
GTAACAGTGGCCTTTATTATTTTCTGGGTGAGGAGTAAGAGGAGCAGGCTCCTGCACAGTGACTACATGAACATGAC
TCCCCGCCGCCCCGGGCCCACCCGCAAGCATTACCAGCCCTATGCCCCACCACGCGACTTCGCAGCCTATCGCTCCT
TCGAACGTTTCTCTGTTGTTAAACGGGGCAGAAAGAAACTCCTGTATATATTCAAACAACCATTTATGAGACCAGTA
CAAACTACTCAAGAGGAAGATGGCTGTAGCTGCCGATTTCCAGAAGAAGAAGAAGGAGGATGTGAACTGGAATTCAG
AGTGAAGTTCAGCAGGAGCGCAGACGCCCCCGCGTACCAGCAGGGCCAGAACCAGCTCTATAACGAGCTCAATCTAG
GACGAAGAGAGGAGTACGATGTTTTGGACAAGAGACGTGGCCGGGACCCTGAGATGGGGGGAAAGCCGAGAAGGAAG
AACCCTCAGGAAGGCCTGTACAATGAACTGCAGAAAGATAAGATGGCGGAGGCCTACAGTGAGATTGGGATGAAAGG
CGAGCGCCGGAGGGGCAAGGGGCACGATGGCCTTTACCAGGGTCTCAGTACAGCCACCAAGGACACCTACGACGCCC
TTCACATGCAGGCCCTGCCCCCTCGC**TGA**GTTTAAACGCGGCCGC

NheI-Kozak-fVII light chain (Start codon + signal + mature fVII light chain)-BamHI*-hIgG1hinge-BamHI*-CD28 (TM+CD)-BstBI-4-1BB-EcoRI-CD3zeta CD-Stop codon-PmeI-NotI

Supplementary Figure S1. The cDNA sequences of TF-targeting CAR1 in pCDH lentiviral vector. A. CAR1 monomer (GenBank accession # MF806378). **B.** CAR1 dimer (GenBank accession # MF806379). TM: Transmembrane domain. CD: Cytoplasmic domain. NheI, BamHI, BstBI, EcoRI, PmeI and NotI: Restriction enzymes are used for molecular subcloning of individual recognition and signaling fragments into plasmid constructs, allowing to further modify and improve CAR signaling fragments, for example, replacing 4-1BB with OX40 (as **TF-targeting CAR2**) or adding OX40 to CAR1 as a fourth generation CAR (as **TF-targeting CAR3**).

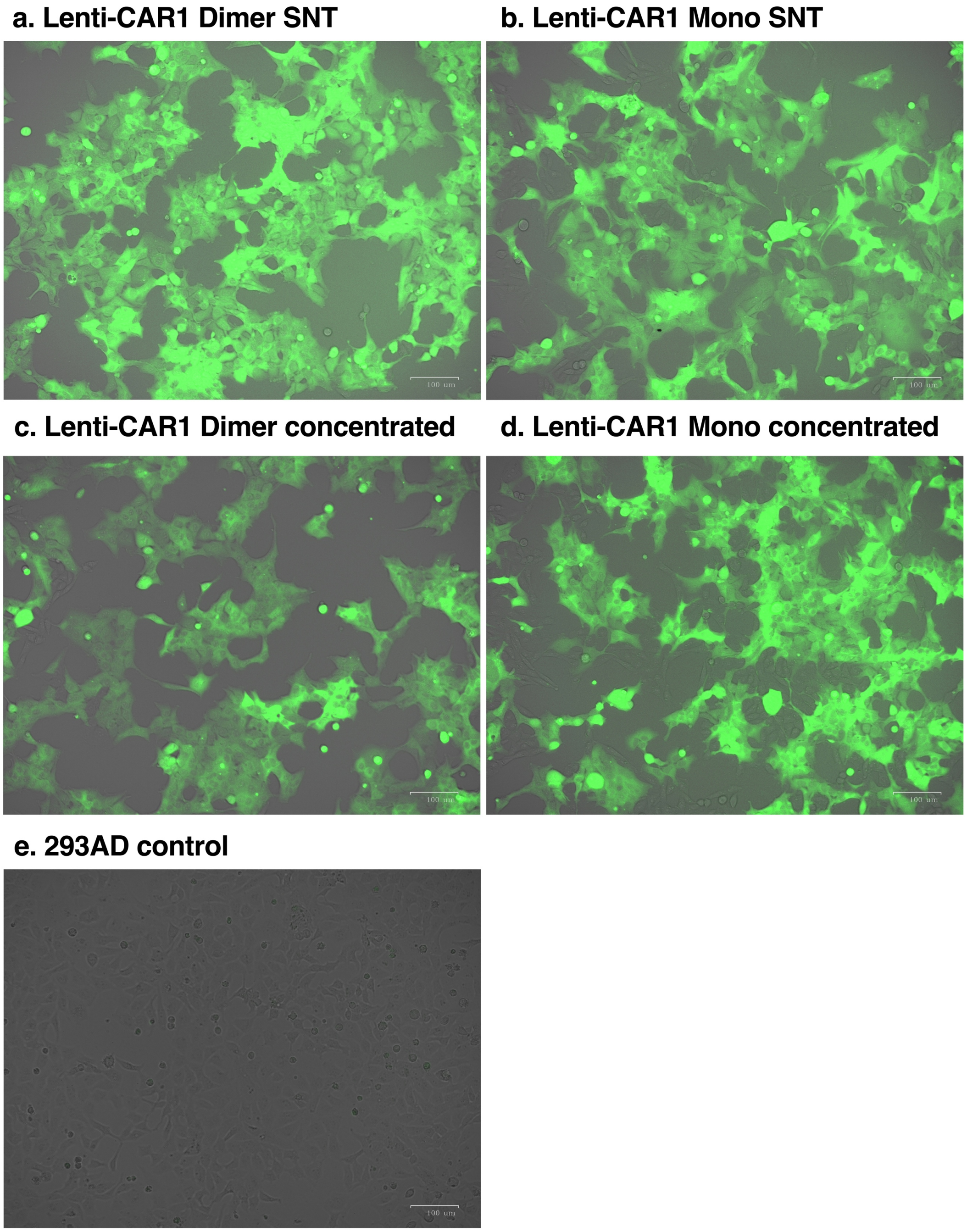

Supplementary Figure S2. Lentivirus encoding TF-targeting CAR1 dimer and monomer, either in unpurified virus in supernatant (SNT) (a, b) or PEG-it purified virus (c, d), can transduce human embryonic kidney cells 293AD. Control 293AD cells were infected by an adenovirus without encoding GFP as a GFP-negative control. Photos were first taken using bright field and green channels and then merged under Zeo Cell Imager (Bio-Rad). Scale bars: 100 μm.

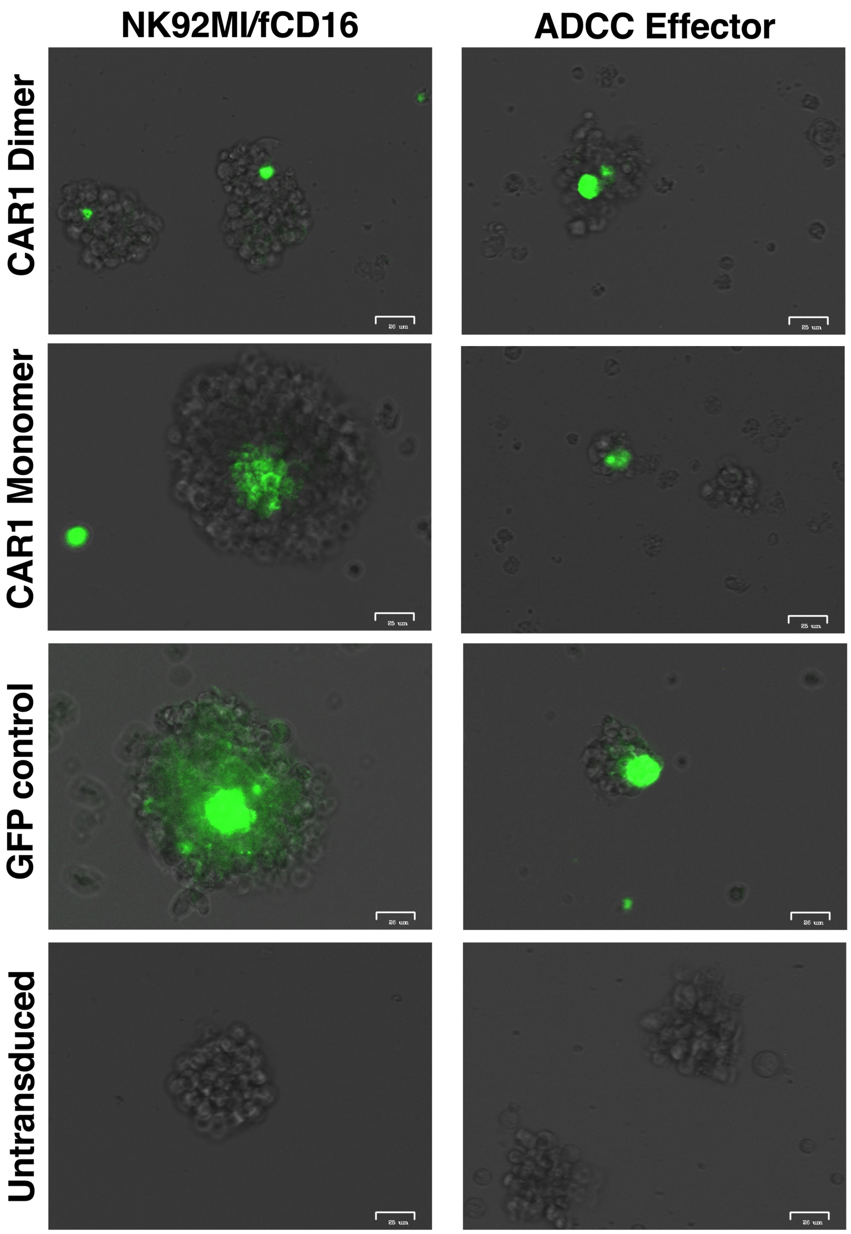

Supplementary Figure S3. Transduction of NK92MI/fCD16 and ADCC Effector cells by lenti-CAR1 monomer, -CAR1 dimer and -GFP. Representative photos of lenti-CAR1 monomer, dimer or GFP-transduced NK92MI/fCD16 cells and ADCC Effector cells (a Jurkat-based T cell line stably transfected with CD16 and N-FAT/Luciferase, Promega) were taken on day 2 post lentivirus transduction and before the addition of puromycin (on day 3). Lentivirus encoding GFP (Lenti-GFP)-transduced cells were used as a lentivirus-transduction control. Untransduced cells did not express GFP as a GFP-negative control. Photos were taken using

bright field and green channels and were then merged under Zeo Cell Imager (Bio-Rad). Scale bars: 25 μm.

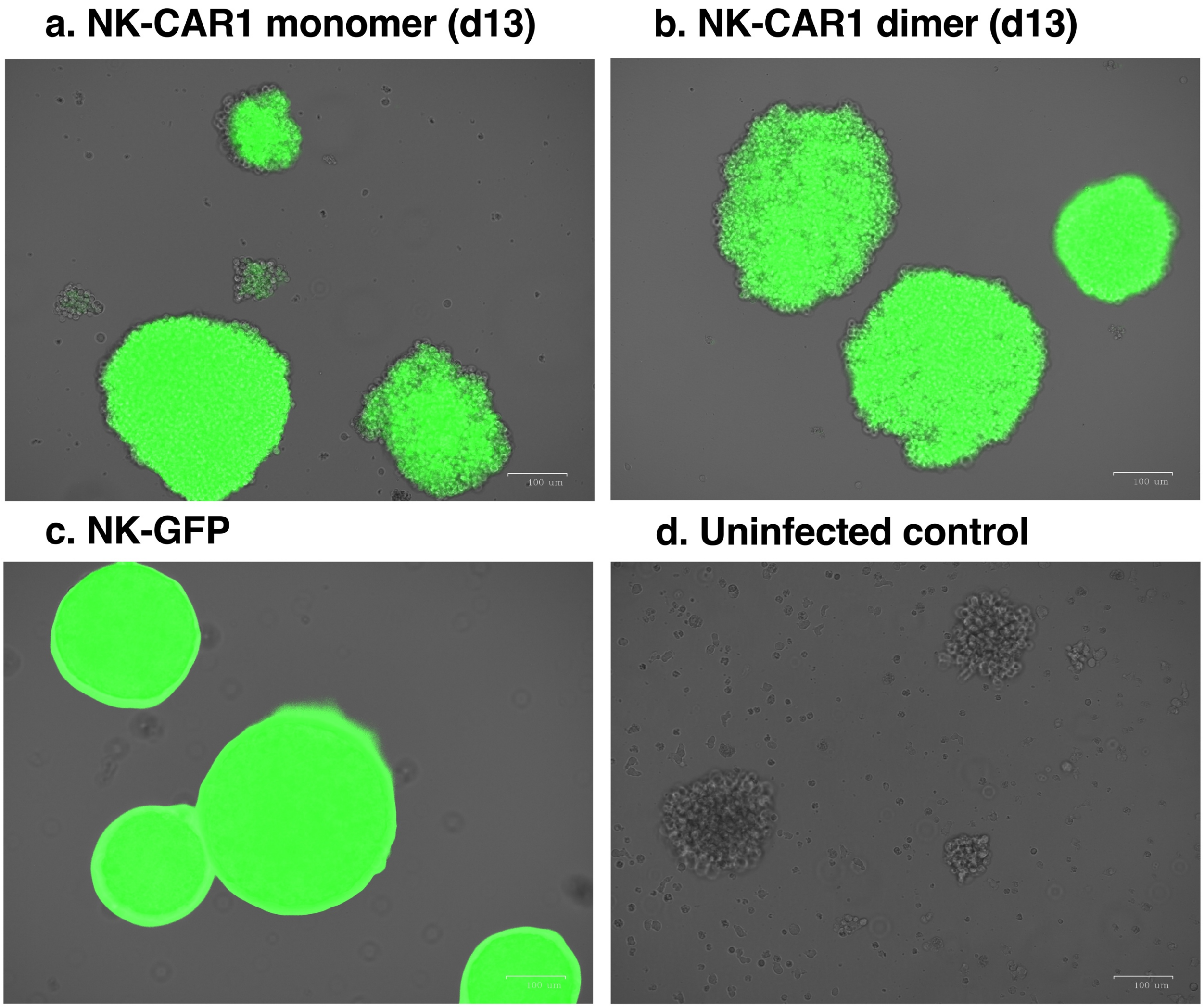

Supplementary Figure S4. Generation of stable TF-targeting CAR1-NK cell lines. a & b. Lenti-CAR1 monomer and dimer-infected NK92MI/fCD16 stable cell lines on day 13 post selection under 5.0 μg/ml puromycin. **c**. Lentivirus encoding GFP (Lenti-GFP)-infected NK92MI/fCD16 cells as a lentivirus-transduced control. **d**. Uninfected NK92MI cells did not express GFP as a GFP-negative control. Photos were taken using bright field and green channels and were then merged under Zeo Cell Imager (Bio-Rad). Scale bars: 100 μm.

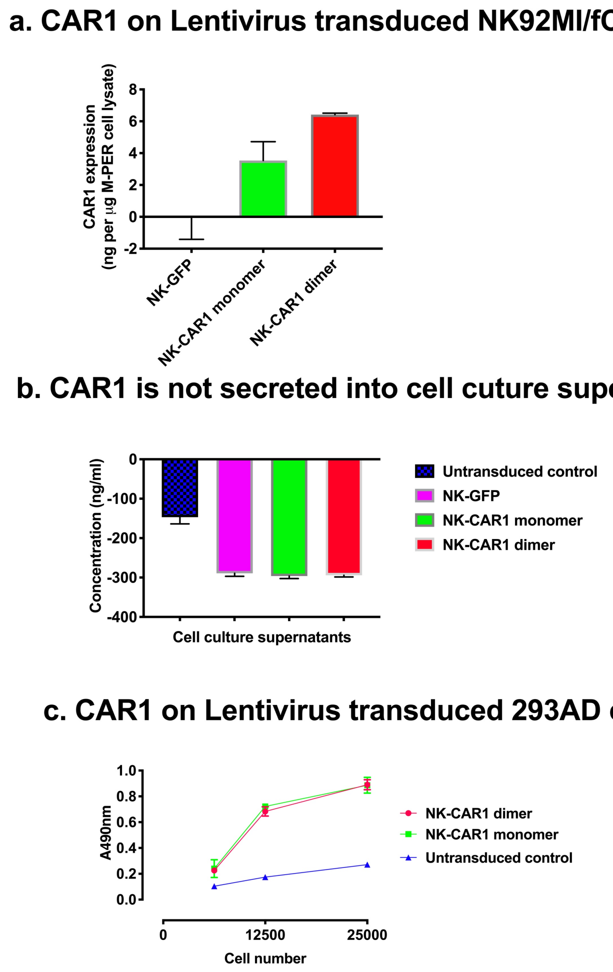

Supplementary Figure S5. Expression of CAR1 on lentivirus infected NK92MI and 293AD cells. a & b. Paired fVII Ab Sandwich ELISA assaying CAR1 expression in M-PER whole cell lysates and in the supernatants of CAR-NK and control cell culture media (day 5). Recombinant L-ICON1 protein was used to generate standard curve. **c**. Cell ELISA assaying CAR1 expression on the cell surface of Lentivirus infected and uninfected 293AD cells. Detection antibody was HRPO conjugated sheep polyclonal antibody against human fVII (Cedarlane Laboratories). Data were analyzed by t test or ANOVA (Prism software) and were presented as mean ± SEM.

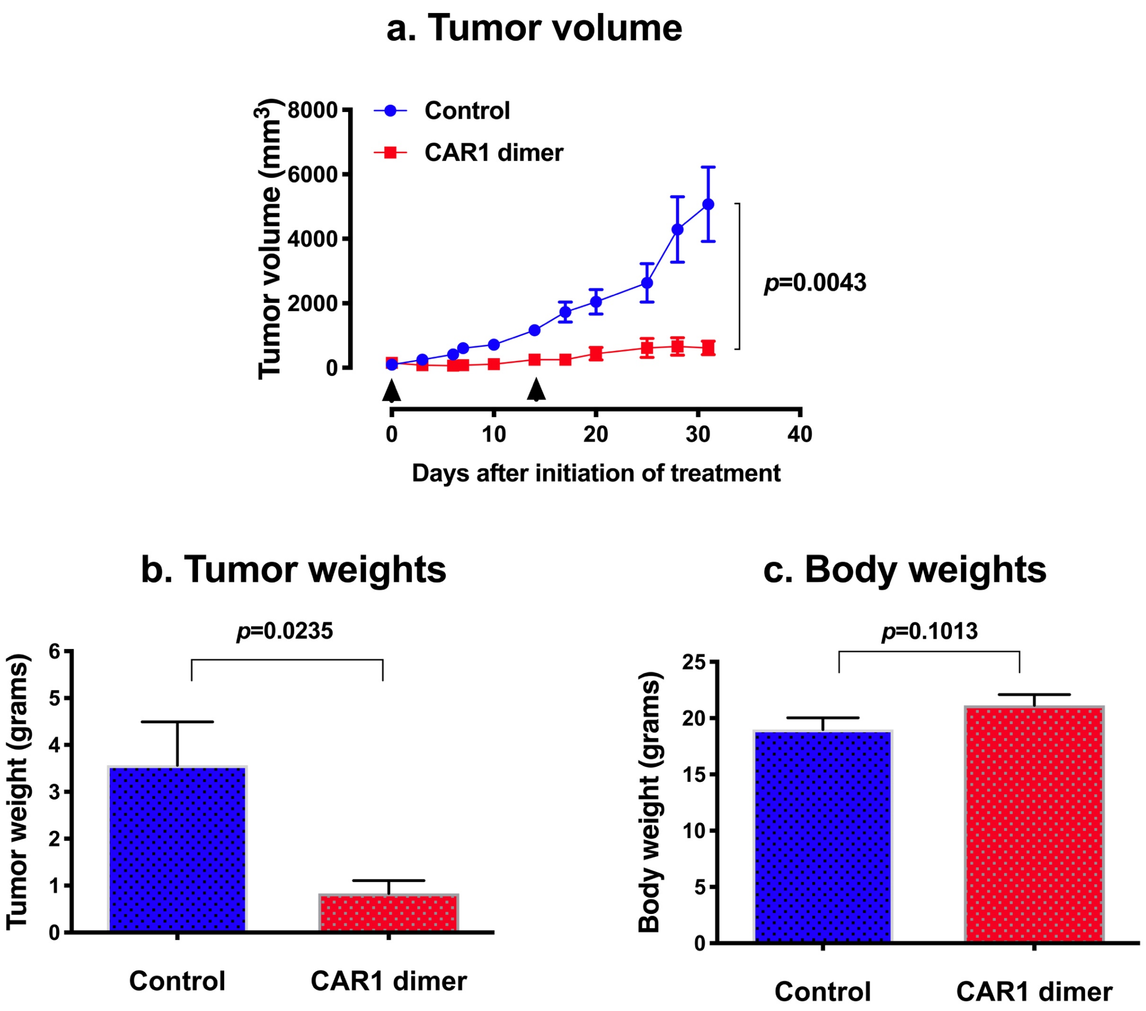

Supplementary Figure S6. TF-CAR-NK cell therapy was effective and safe in a pilot study for the treatment of human TNBC PDX in an orthotopic SCID mouse model. TNBC PDX (JAX) was generated in four-week old female CB-17 SCID mice. When the PDX tumors reached ~100 mm^3, NK-CAR1 dimer (NK92MI/fCD16/CAR1 dimer) or NK92MI/fCD16 cells without CAR constructs (as a control) were injected i.v. via tail veins two times (5×10^6 cells per mouse on day 0 and 3×10^6 cells per mouse on day 14, arrows) (three mice per group). **a**. Tumor volumes. Tumor (**b**) and net mouse body (**c**) weights were measured at the time of sacrifice of animals. P values were analyzed using Prism Software.

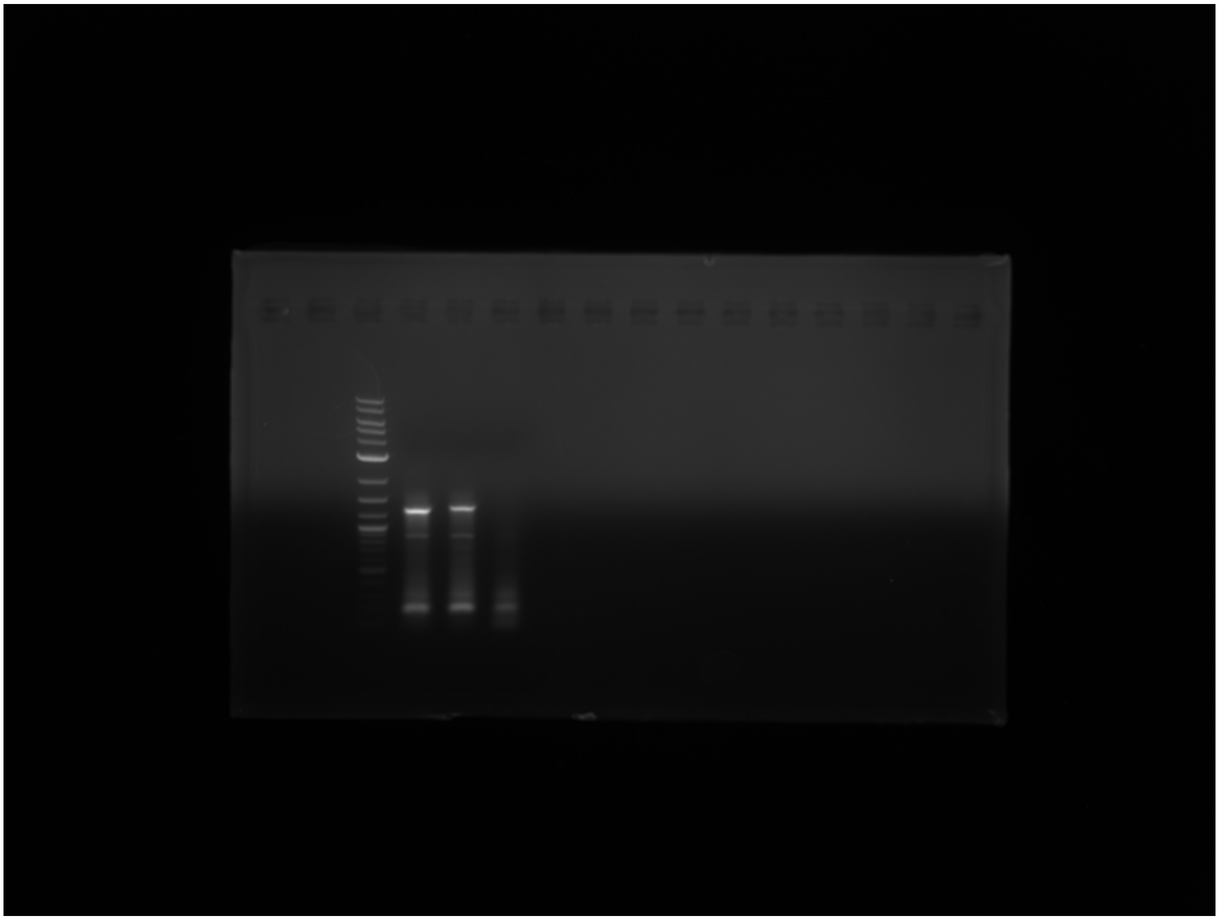

Supplementary Figure S7. RT-PCR for TF-CAR cDNAs in NK-CAR1 monomer, dimer and uninfected NK92MI parental control. Total RNA was extracted by Trizol reagents from NK92MI/fCD16 cells stably transfected with CAR1 monomer (M) or dimer (D). Parental NK92MI cells were used as a negative control (C) for CAR1 (hfVII light chain with or without IgG1 hinge followed by CD28, 4-1BB and CD3 zeta). Samples from left to right:

a. DNA ladder (Quick-Load 2-Log DNA ladder, New England BioLabs)
b. NK-CAR1 monomer
c. NK-CAR1 dimer
d. Parental NK92MI as a negative control.

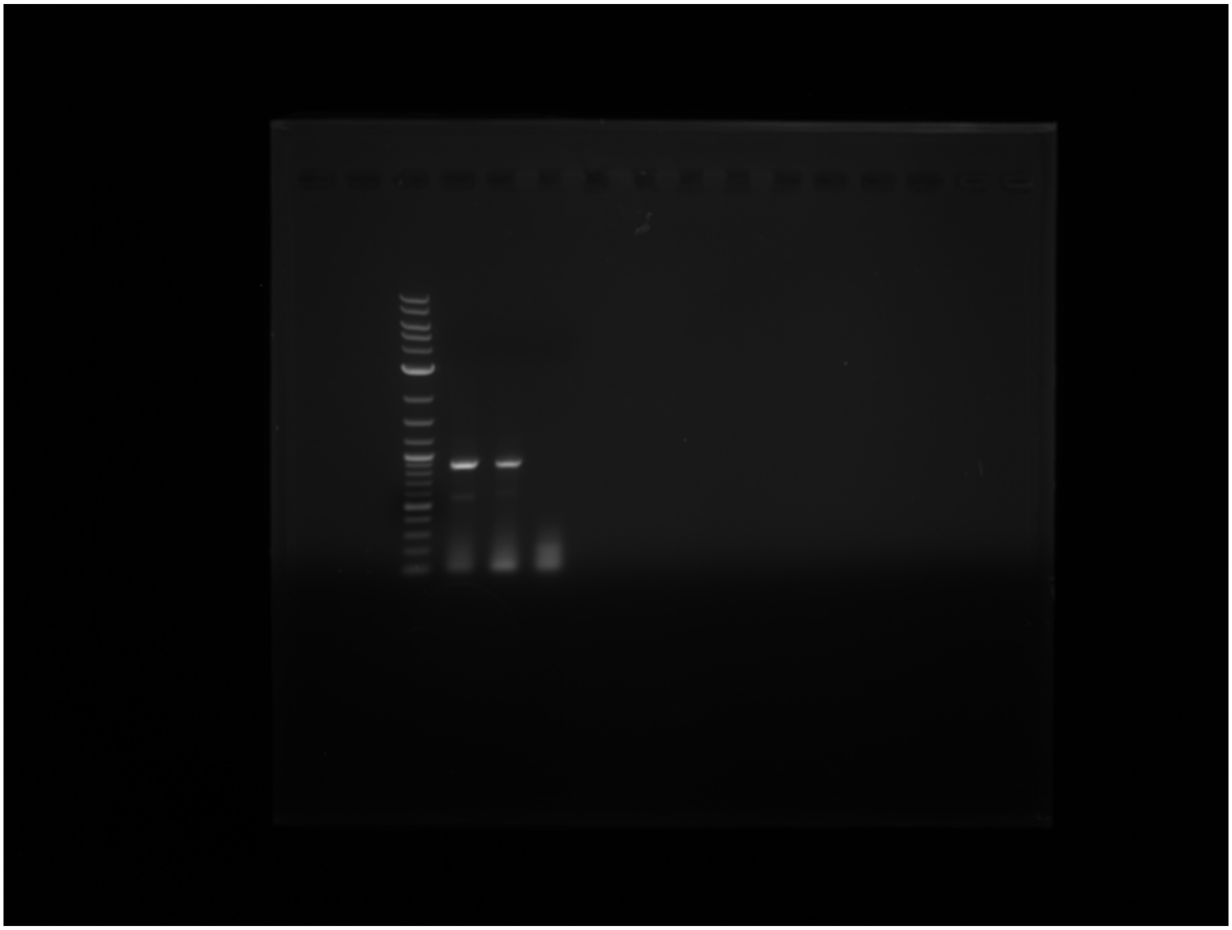

Supplementary Figure S8. RT-PCR for CD16 cDNA in NK-CAR1 monomer, dimer and uninfected NK92MI parental control. Total RNA was extracted by Trizol reagents from NK92MI/fCD16 cells stably transfected with CAR1 monomer (M) or dimer (D). Parental NK92MI cells were used as a negative control (C) for CD16. Samples from left to right:

a. DNA ladder (Quick-Load 2-Log DNA ladder, New England BioLabs)
b. NK-CAR1 monomer
c. NK-CAR1 dimer
d. Parental NK92MI as a negative control.

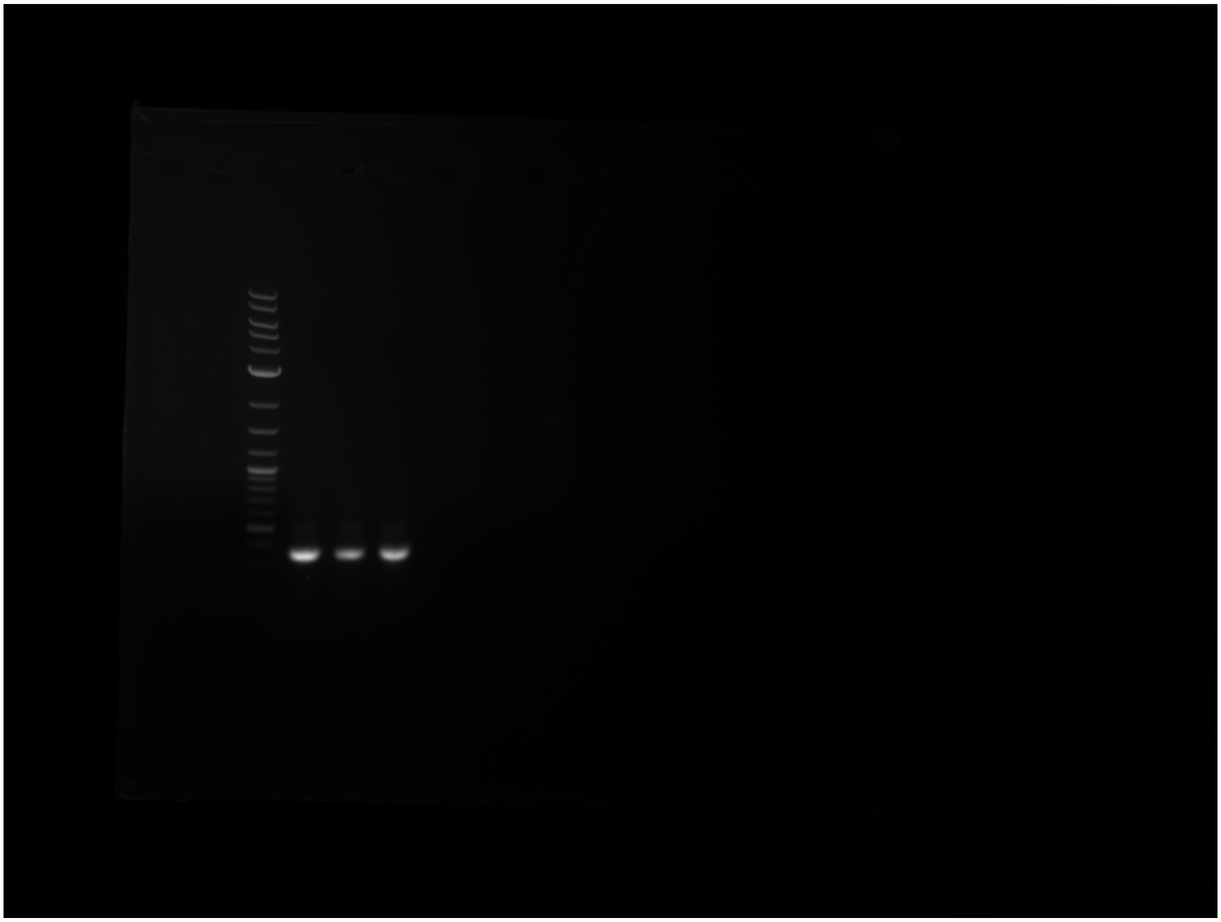

Supplementary Figure S9. RT-PCR for CD16 cDNA in NK-CAR1 monomer, dimer and uninfected NK92MI parental control. Total RNA was extracted by Trizol reagents from NK92MI/fCD16 cells stably transfected with CAR1 monomer (M) or dimer (D). Parental NK92MI cells were used as a negative control (C) for beta actin as a loading control for CAR1 and CD16. Samples from left to right:

a. DNA ladder (Quick-Load 2-Log DNA ladder, New England BioLabs)
b. NK-CAR1 monomer
c. NK-CAR1 dimer
d. Parental NK92MI as a negative control.

Supplementary Table 1. Primers for RT-PCR

cDNA	Primer name	Primer sequence
CAR1	5'-hfVII	GCTAGCGCCACCATGGTCTCCCAGGCCCTC
	3'-CD3zeta	GCGGCCGCGTTTAAACTCAGCGAGGGGGCAGGGCCTGCAT
CD16	5'-CD16	ACGATCTTAAGCTTGCCGCCACCATGGGTGGAGGGGCTG
	3'-CD16	AGTTTA GCGGCCGCTTATCATTTGTCTTGAGGGT
β actin	5'-actin	TGGAATCCTGTGGCATCCATGAAAC
	3'-actin	TAAAACGCAGCTCAGTAACAGTCCG

Supplementary video (Separately attached, labeled as Video #1-#4)

Video #1: TF-CAR-NK cells (CAR1-NK monomer, green) can kill human TNBC (MDA-MB-231, adherent) cells

Video #2: GFP-NK control cells have no killing activity to human TNBC

Video #3: TF-CAR-NK cells (CAR1-NK monomer, green) can kill murine TNBC (4T1, adherent) cells

Video #4: GFP-NK control cells have no killing activity to murine TNBC (4T1)
